# Protective effects of calcyclin-binding protein against pulmonary vascular remodeling in flow-associated pulmonary arterial hypertension

**DOI:** 10.1186/s12931-022-02137-z

**Published:** 2022-08-30

**Authors:** Jingjing Zhou, FuRong Li, Yicheng Yang

**Affiliations:** 1 Echocardiography Medical Center, Maternal-Fetal Medicine Center in Fetal Heart Disease, Beijing Anzhen Hospital, Capital Medical University, Beijing, China; 2 Department of Laboratory Medicine, Shanghai University of Medicine and Health Sciences Affiliated Zhoupu Hospital, Shanghai, China; 3 Center of Pulmonary Vascular Disease, State Key Laboratory of Cardiovascular Disease, National Center for Cardiovascular Disease, Fuwai Hospital, Chinese Academy of Medical Sciences and Peking Union Medical College, North Lishi Road, Xicheng, No. 167, Beijing, 100037 China

**Keywords:** Congenital heart disease, Pulmonary arterial hypertension, Pulmonary artery smooth muscle cells, Calcyclin-binding protein/Siah-1-interacting protein, Pulmonary vascular remodeling

## Abstract

**Background:**

Pulmonary arterial hypertension associated with congenital heart disease (CHD-PAH) is recognized as a cancer-like disease with a proliferative and pro-migratory phenotype in pulmonary artery smooth muscle cells (PASMCs). Calcyclin-binding protein/Siah-1-interacting protein (CacyBP/SIP) has been implicated in the progression of various cancers; however, it has not been previously studied in the context of CHD-PAH. Here, we aimed to examine the function of CacyBP/SIP in CHD-PAH and explore its potential as a novel regulatory target for the disease.

**Methods:**

The expression of CacyBP/SIP in PASMCs was evaluated both in the pulmonary arterioles of patients with CHD-PAH and in high-flow-induced PAH rats. The effects of CacyBP/SIP on pulmonary vascular remodeling and PASMC phenotypic switch, proliferation, and migration were investigated. LY294002 (MedChemExpress, NJ, USA) was used to block the phosphoinositide 3-kinase/protein kinase B (PI3K/AKT) pathway to explore changes in PASMC dysfunction induced by low CacyBP/SIP levels. Hemodynamics and pulmonary arterial remodeling were further explored in rats after short-interfering RNA-mediated decrease of CacyBP/SIP expression.

**Results:**

CacyBP/SIP expression was markedly reduced both in the remodeled pulmonary arterioles of patients with CHD-PAH and in high-flow-induced PAH rats. Low CacyBP/SIP expression promoted hPASMC phenotypic switch, proliferation, and migration via PI3K/AKT pathway activation. Our results indicated that CacyBP/SIP protected against pulmonary vascular remodeling through amelioration of hPASMC dysfunction in CHD-PAH. Moreover, after inhibition of CacyBP/SIP expression in vivo, we observed increased right ventricular hypertrophy index, poor hemodynamics, and severe vascular remodeling.

**Conclusions:**

CacyBP/SIP regulates hPASMC dysfunction, and its increased expression may ameliorate progression of CHD-PAH.

**Supplementary Information:**

The online version contains supplementary material available at 10.1186/s12931-022-02137-z.

## Background

Pulmonary arterial hypertension (PAH) is a relatively common complication of congenital heart disease (CHD), which manifests as an abnormal elevation in pulmonary arterial pressure and increased vascular resistance, leading to heart failure and eventually death [1–3]. Typically, the pathophysiology of PAH is characterized by progressive remodeling of small pulmonary arteries and muscularization of precapillary arterioles attributed to a neoplastic-like proliferative and pro-migratory phenotype of pulmonary smooth muscle cells (PASMCs) [4, 5]. CHD-PAH with left-to-right shunt embraces a distinct reversible phase, indicating that timely surgical repair of congenital lesions generally restores normal pulmonary blood flow and reverses pulmonary arteriopathy [6]. However, the beneficial effects of shunt closure are later lost, and pathologic pulmonary vascular remodeling persists or even aggravates after surgery [7]. These observations emphasize the crucial significance of early and accurate determination of this “reversible phase” in patients with CHD-PAH, which is still poorly understood. Further investigation of molecular factors implicated in pulmonary vascular remodeling may provide insight into the elucidation of the underlying mechanisms and identify the “reversible phase”. Given the overlap with neoplastic-like processes, potential targets discovered in cancer may lead to an emerging paradigm in CHD-PAH pathology, potentially providing inspiring therapeutic strategies to reverse established pulmonary vascular remodeling in CHD-PAH patients.

Calcyclin-binding protein or Siah-1-interacting protein (CacyBP/SIP) is involved in multiple cellular processes, including ubiquitination, proliferation, apoptosis, differentiation, tumorigenesis, and regulation of transcription by interacting with target proteins [8–11]. Essential regulatory roles for CacyBP/SIP in various cancers have been determined [12–16], and CacyBP/SIP has been regarded as a potential target for cancer therapy. However, the function of CacyBP/SIP in PAH, known as a cancer-like disease, remains to be explored.

Therefore, we aimed to investigate the exact role and underlying molecular mechanisms of CacyBP/SIP in CHD-PAH. Here, we examined the expression of CacyBP/SIP in the pulmonary arterioles of patients with CHD-PAH and in flow-associated PAH rats along with the effects of downregulation and upregulation of CacyBP/SIP expression on human PASMC (hPASMC) function. Moreover, adeno-associated viruses expressing short interfering CacyBP/SIP (AAVs-siCacyBP) were used to reduce CacyBP/SIP expression in vivo to further examine relevant phenotypes on hemodynamics and morphology.

## Methods

### Collection of patients’ lung tissue

The procedures performed adhered to the tenets of the Declaration of Helsinki and were approved by the Ethics Committee of Fuwai Hospital. We enrolled 22 patients with CHD-PAH who underwent repair surgery, and classified them into reversible and irreversible groups after 1 year follow-up, as described in our previous study [17]. Lung tissues from CHD-PAH patients were collected to explore expression of CacyBP/SIP. Normal lung tissues from 12 patients with early-stage lung cancer were used as control. All participants involved in this study provided written informed consent.

### Flow-associated PAH rat model

Male Sprague–Dawley (SD) rats (270–290 g, 7 weeks old), provided by the Beijing Vital River Laboratory Animal Technology Co. Ltd., were housed in a specific pathogen-free environment and maintained on a standard rat diet, under a 12-h light/dark cycle and constant temperature (22–24℃) and humidity (45–55%) in the State Key Laboratory of Cardiovascular Disease (Beijing, China). After a 1-week adaptation period, rats were randomly divided into three groups: (1) control (n = 6), (2) sham (n = 6), and (3) flow-associated PAH (n = 8). Monocrotaline (MCT, Sigma-Aldrich, USA, 60 mg/kg) injection followed by abdominal aortocaval shunt (AV) was used to generate flow-associated PAH in rats, as described previously [17, 18]. Rats underwent right heart catheterization to obtain hemodynamic parameters including right ventricular systolic pressure (RVSP), pulmonary arterial systolic pressure (PASP), and mean pulmonary arterial pressure (mPAP) after 4 weeks feeding [19]. All animal experiments were conducted in accordance with the guidelines of the Institutional Animal Care and Use Committee (IACUC). Right ventricular hypertrophy (RVH) was evaluated using the RVH index (also called Fulton index) [RVHI, calculated as the weight ratio of RV/(left ventricle plus atrial ventricular septum)]. Fresh right lung tissues were frozen in liquid nitrogen for western blotting, while the left lungs were fixed with 4% paraformaldehyde and embedded in paraffin for morphological examination.

### Transduction with adeno-associated viruses (AAVs) in rats

Animal experiments were also conducted to verify the effects of low CacyBP/SIP expression on hemodynamics and morphology in vivo. AAVs inhibiting CacyBP/SIP (AAV-siCacyBP) expression were purchased from Hanbio Biotechnology Co., Ltd. (Shanghai, China) and used to decrease CacyBP/SIP expression in pulmonary arterioles. The AAV9 vector (GV478: U6-MCS-CAG-EGFP) contains a U6 promoter-driven siRNA expression system. An intratracheal one-time instillation of AAVs (1.5 × 10^12^ transducing units) or PBS was delivered to SD male rats (7 weeks old, 270–290 g) after pentobarbital anesthesia (50 mg/kg intraperitoneally). Several rats were randomly selected and euthanized at 1 week to obtain lung slices to evaluate transduction efficiency, based on the intensity of adenoviral-expressed green fluorescent protein (GFP) signal observed with a fluorescent confocal microscope. The remaining AAV-transfected rats were used to induce flow-associated PAH.

### Morphometric analysis of pulmonary arteries

Paraffin-embedded lung tissues were cut into 5-μm thick sections. Lung slices were stained with hematoxylin and eosin (H&E) and elastic van Gieson (EVG) staining for morphometric analysis. The pathological grade of the pulmonary arterioles was determined according to the standard of Heath-Edwards Classification [20]. Peripheral vessels 50–150 µm in diameter were selected and examined. The ratios of pulmonary vascular wall thickness/vascular external diameter (WT%) and pulmonary vascular wall area/total pulmonary vascular area (WA%) were calculated to evaluate pulmonary vascular remodeling (10–15 arterioles were calculated per rat).

### Immunohistochemical (IHC) staining

After dewaxing in water, 3% H_2_O_2_ was used to block endogenous peroxidase activity in the lungs. Slices were then incubated with various primary antibodies (Additional file 1: Table S1) at 4 °C overnight. After incubation with horseradish peroxidase (HRP)-labeled secondary antibodies, samples were treated with diaminobenzidine and counterstained with hematoxylin. Stained sections were visualized under a microscope. Average optical density (AOD) was used to evaluate expression of the targeted proteins with ImageJ.

### Immunofluorescent (IF) staining

After blocking with goat serum, lung slices or PASMCs were incubated with diluted specific primary antibodies (Additional file 1: Table S1) at 4 °C overnight. Samples were then incubated with fluorescently labeled secondary antibodies and 4′6-diamidino-2-phenylindole (DAPI) at room temperature for 1 h. A confocal fluorescence microscope (Leica TCS SP2, Germany) was used to observe and record images.

### Isolation, culture, and identification of rat PASMCs

Pulmonary arteries from control, sham, and MCT-AV groups were used to isolate PASMCs as described previously [21]. PASMC purity was confirmed with IF staining for alpha-smooth muscle actin (α-SMA) and platelet endothelial cell adhesion molecule-1 (CD31).

### Cell culture and adenovirus transduction of hPASMCs

hPASMCs were purchased from Sciencell Research Laboratories (Cat#3110), and cultured in smooth muscle cell medium (Cat#1101, Sciencell Research Laboratories, USA) supplemented with 2% fetal bovine serum. Cells were transduced with Adv-siCacyBP (or Adv-CacyBP), or the control adenovirus Adv-GV119 (or Adv-GV314) (Hanbio Biotechnology Co., Ltd., Shanghai, China) at a multiplicity of infection for 10 h. After 72 h, efficiency of transductions was verified using western blot analysis.

### Proliferation assays

Cell proliferation was evaluated using 5-ethynyl-2-deoxyuridine (EdU) labeling and cell counting assays. The BeyoClick™ EdU-594 Cell Proliferation Detection Kit (Cat#C0078S, Beyotime, China) was used. Transfected hPASMCs were plated in six-well plates for 36 h, and the preheated EdU working solution was added to each well. After incubation for 2 h, EdU-labeled cells were fixed with 4% paraformaldehyde for 20 min and subsequently incubated with 0.3% TritonX-100 for 20 min and click reaction solution for 30 min. Hoechst 33,342 solution was then added to label the nuclei of living cells. Images were acquired using fluorescence microscopy.

For the cell count assay, transduced hPASMCs were plated at a density of 2.5 × 10^5^ cells per well in a six-well plate. After incubation for 36 h, cells were harvested with trypsin–EDTA, and counted using 0.4% trypan blue with a hemocytometer.

### Migration assays

Cell migration was evaluated with wound healing and transwell assays. For wound healing experiments, transduced hPASMCs (at a density of 2 × 10^5^ cells/well) were plated in six-well plates until they reached confluence. A linear wound was gently created using a 200 µL yellow micropipette tip, and the medium was replaced with serum-free smooth muscle cell medium. The wound healing process was photographed using an inverted microscope. Wound healing was quantified by calculating the percentage of closed scratches [(initial scratch width – final scratch width) /initial scratch width × 100%] [22].

For transwell assays, transduced hPASMCs (at a density of 10^4^ cells/well) were seeded into the upper part of the transwell chamber (Corning Ltd., USA), and smooth muscle cell medium containing 2% fetal bovine serum was added to the lower chamber. After 16 h, the cells remaining on the upper surface of the filter were gently removed with cotton swabs, and the cells that migrated to the lower surface were stained with 0.1% crystal violet. Migratory cells were counted five times in random fields of view under a microscope.

### Western blotting

Samples were homogenized in radioimmunoprecipitation assay (RIPA) buffer containing proteinase and phosphatase inhibitors. The supernatant was collected for total protein measurement using a BCA kit (Beyotime Biotechnology, China). Extracted proteins were separated with sodium dodecyl sulfate–polyacrylamide gel electrophoresis (SDS-PAGE) and transferred to a polyvinylidene fluoride membrane. The membranes were incubated overnight at 4 °C with specific primary antibodies (Additional file 1: Table S1). Subsequently, the membranes were incubated with appropriate secondary antibodies for 1 h, and proteins were visualized using an enhanced chemiluminescence (ECL) kit (Millipore, USA). Semi-quantitative densitometric analyses were performed with ImageJ. GAPDH was used as the protein loading control.

### Statistical analysis

All statistical analyses were conducted using SPSS statistics software (version 24.0; IBM Corp, Chicago, IL, United States). The Shapiro–Wilk test was used to assess normality of the data distribution. For normally distributed data, student’s t-test or one-way analysis of variance (ANOVA) followed by Tukey’s multiple comparison test was used according to equal variance. If the assumption of homogeneity of variance was violated, Welch’s t-test or Welch’s ANOVA was applied. The non-parametric test was used for comparisons of non-normally distributed data. Correlations between CacyBP/SIP expression and the pathological grade of pulmonary arterioles were assessed using Spearman’s correlation (two-tailed) analyses. For all analyses, differences were considered statistically significant if two-sided *P* was < 0.05.

## Results

### Decreased expression of CacyBP/SIP in the pulmonary arterioles of patients with CHD-PAH

**Fig. 1:**
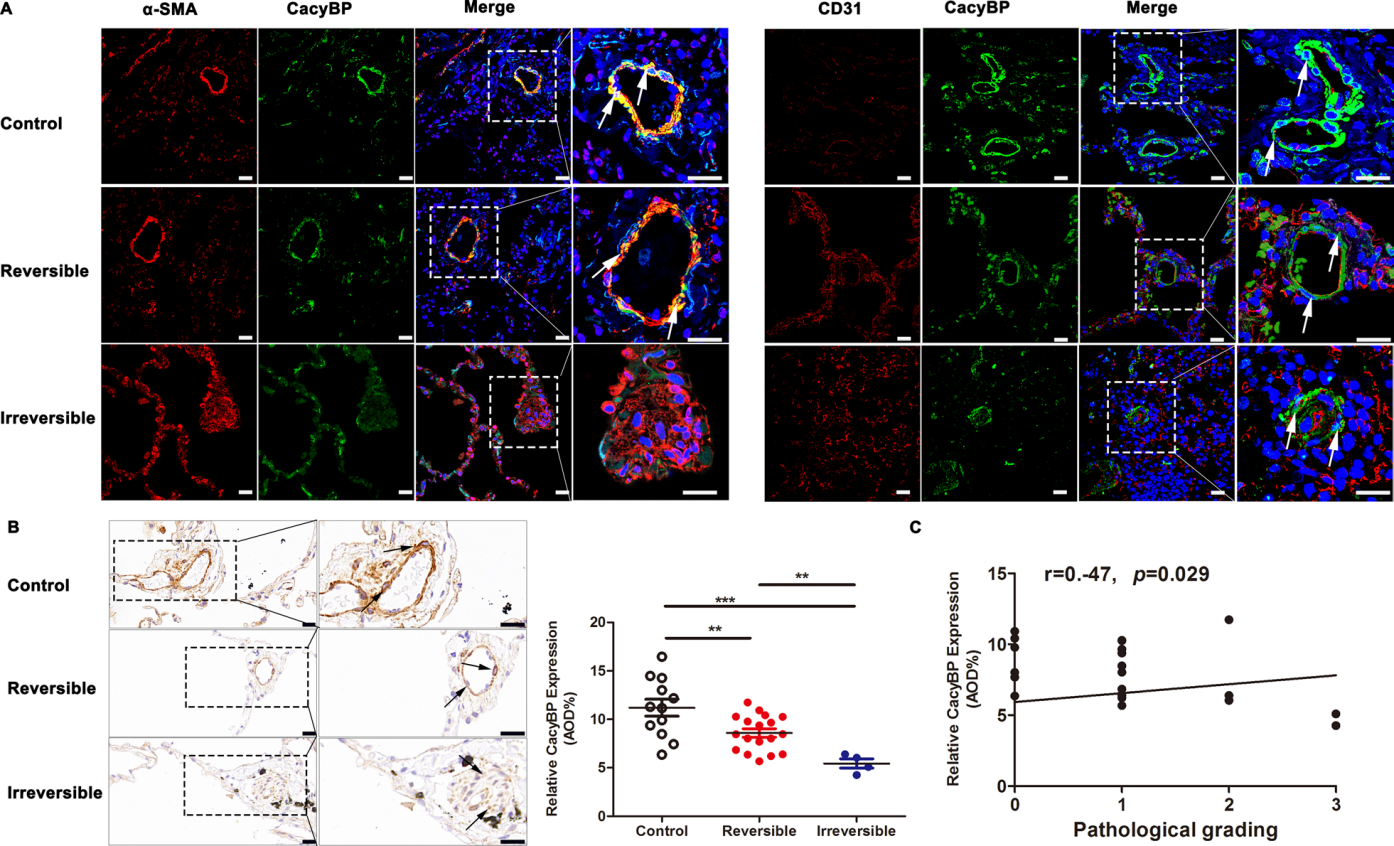
CacyBP/SIP expression was significantly decreased in pulmonary arterioles of patients with CHD-PAH. **a** Representative confocal images of double-label IF staining of CacyBP/SIP and α-SMA (or CD31) in the pulmonary arterioles of CHD-PAH patients. Size bars, 25 µm. **b** Representative IHC images and analysis of CacyBP/SIP expression in the pulmonary arterioles of patients. Scale bars, 20 µm. AOD, calculated as the integrated option density/area, was used to quantify expression of CacyBP. Data are shown as mean ± SEM (normal control group: n = 12; reversible group: n = 18; irreversible group: n = 4). **P < 0.01, ***P < 0.001. **c** Correlation between CacyBP/SIP expression and the pathological grade of pulmonary arterioles in patients with CHD-PAH (n = 22). *IF* immunofluorescent, *IHC* immunohistochemistry, *CacyBP/SIP* calcyclin-binding protein, *α-SMA* alpha smooth muscle actin, *CD31* platelet endothelial cell adhesion molecule-1, *CHD* congenital heart disease, *PAH* pulmonary arterial hypertension, *AOD* average optical density

Expression of CacyBP/SIP in the pulmonary arterioles of patients with CHD-PAH was assessed. CacyBP/SIP was mainly expressed in α-SMA-positive PASMCs, while the levels of CacyBP/SIP were lower in the pulmonary arterioles of patients with CHD-PAH, especially in the irreversible group, than in normal controls (Fig. 1A and B). In addition, as shown in Fig. 1C, CacyBP/SIP expression was negatively correlated with the pathological grade of the pulmonary arterioles. These findings indicated that the decrease of CacyBP/SIP expression may be associated with advanced pulmonary vascular remodeling in CHD-PAH.

### Decreased expression of CacyBP/SIP in the pulmonary arterioles of flow-associated PAH rats

**Fig. 2:**
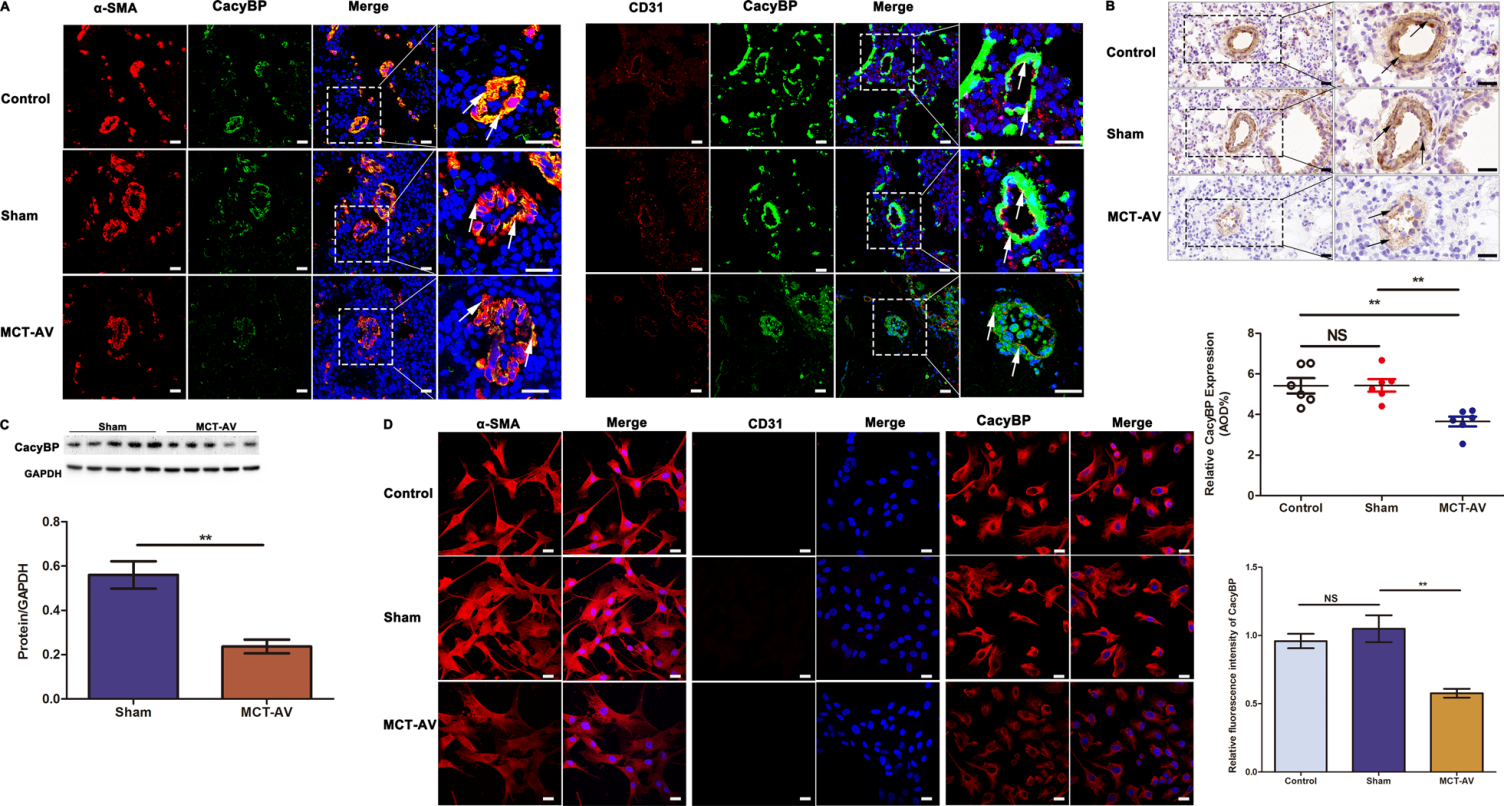
CacyBP/SIP expression was significantly decreased in pulmonary arterioles of flow-associated PAH rats. **a** Representative confocal images of double-label IF staining with CacyBP/SIP and α-SMA (or CD31) in the pulmonary arterioles. Size bars, 25 µm. **b** Representative IHC images and analysis of CacyBP/SIP expression in the pulmonary arterioles. Scale bars, 20 µm. AOD, calculated as the integrated option density/area, was used to quantify the expression of CacyBP. Data are shown as mean ± SEM (n = 6 for each group). NS = no significance, **P < 0.01, ***P < 0.001. **c** Expression of CacyBP/SIP in the lung tissues. Western blots were quantified by calculating the band density and then normalized to GADPH. Data are shown as mean ± SEM (n = 6 for each group). **P < 0.01. **d** Representative IF staining confocal images of α-SMA (or CD31or CacyBP/SIP) in PASMCs and analysis of CacyBP/SIP expression. Size bars, 25 µm. *IF* immunofluorescent, *IHC* immunohistochemistry, *CacyBP/SIP* calcyclin-binding protein, *α-SMA* alpha smooth muscle actin, *CD31* platelet endothelial cell adhesion molecule-1, *MCT* monocrotaline, *AV* aortocaval, *PAH* pulmonary arterial hypertension, *AOD* average optical density

A flow-associated PAH rat model generated with MCT and AV treatment was used to further examine the pattern of CacyBP/SIP expression in pulmonary arterioles. Consistent with our previous findings, expression of CacyBP/SIP was decreased in PAH rats examined with IHC and western blotting (Fig. 2A–C). Moreover, PASMCs were isolated and characterized using IF staining with CD31 and α-SMA antibodies to determine the purity of the cells. We showed that expression of CacyBP/SIP was significantly downregulated in PASMCs from flow-associated PAH rats.

### Reduced CacyBP/SIP expression promoted proliferation and migration of hPASMCs

**Fig. 3:**
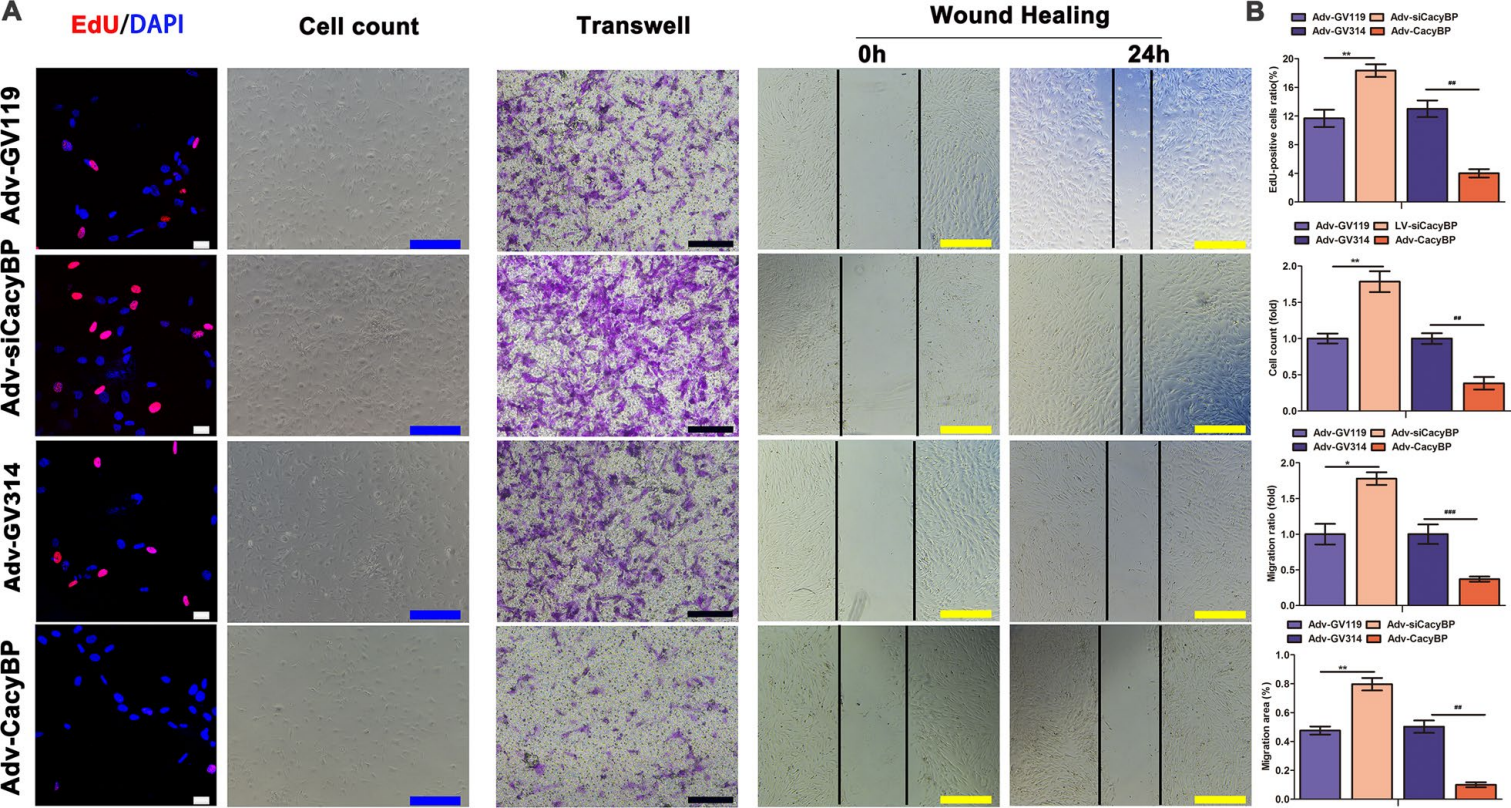
CacyBP/SIP regulated hPASMC proliferation and migration. **a** After transfection with Adv-siCacyBP, proliferation and migration of hPASMCs were enhanced. Scale bars (white: 25 µm; blue: 500 µm; black: 200 µm; yellow: 500 µm). **b** Summary of EdU, cell count, wound healing, and transwell assays in the indicated groups. Data are shown as mean ± SEM (n = 3 for each group). Differences were compared between the Adv-siCacyBP and Adv-GV119 groups, and between the Adv-CacyBP and Adv-GV314 groups. *Adv-siCacyBP vs Adv-GV119; #Adv-CacyBP vs Adv-GV314. *P < 0.05, **P < 0.01; ##*P* < 0.01, ###*P* < 0.001. *EdU* 5-ethynyl-2’-deoxyuridine, *hPASMC* human pulmonary artery smooth muscle cell, *CacyBP/SIP* calcyclin-binding protein

After inhibiting expression of CacyBP/SIP with AdV-siCacyBP transfection, EdU and cell count assays showed that the number of hPASMCs increased significantly compared to that of controls. Moreover, the migratory ability of hPASMCs was exaggerated in hPASMCs transduced with Adv-siCacyBP. Conversely, hyperproliferation and excessive migration of hPASMCs were reversed after CacyBP/SIP overexpression (Fig. 3).

### Decreased CacyBP/SIP expression promoted the phenotypic switch of hPASMCs

**Fig. 4:**
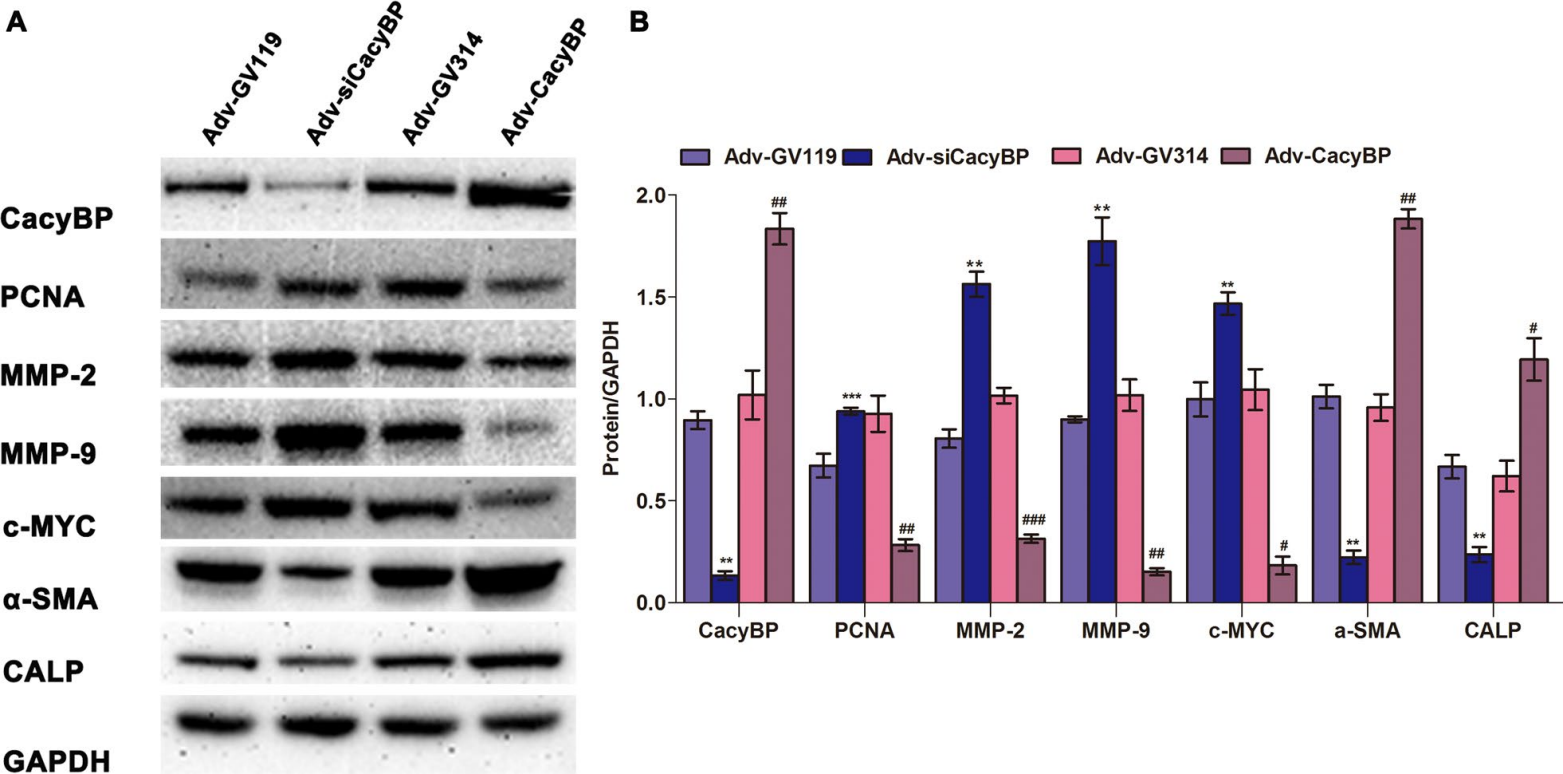
CacyBP/SIP regulated hPASMC phenotypic switch. **a** Expression of CacyBP/SIP and phenotype markers with western blotting. **b** Quantification of protein expression in hPASMCs. Western blots were quantified by calculating the band density normalized to GADPH. Data are shown as mean ± SEM (n = 3 for each group). The difference in the expression of each protein was compared between Adv-siCacyBP and Adv-GV119 groups, and between Adv-CacyBP and Adv-GV314 groups. *Adv-siCacyBP vs. Adv-GV119; #Adv-CacyBP vs Adv-GV314. **P < 0.01, ***P < 0.001; #*P* < 0.05, ##*P* < 0.01, ###*P* < 0.001. hPASMC, human pulmonary artery smooth muscle cell; CacyBP/SIP, calcyclin-binding protein

We attempted to explore how decreased CacyBP/SIP expression promoted proliferation and migration of hPASMCs. As shown in Fig. 4, contractile proteins such as α-SMA and calponin (CALP) were downregulated, while synthetic proteins, including proliferating cell nuclear antigen (PCNA), matrix metalloproteinase 2 (MMP-2), MMP-9, and c-MYC were upregulated after inhibition of CacyBP/SIP expression. Taken together, we showed that the downregulation of CacyBP/SIP in hPASMCs may promote cell phenotypic switching, thus leading to excessive proliferation and migration.

### CacyBP/SIP modulated phenotypic switch of hPASMC via the phosphoinositide 3-kinase (PI3K)/protein kinase B (AKT) signaling pathway

**Fig. 5:**
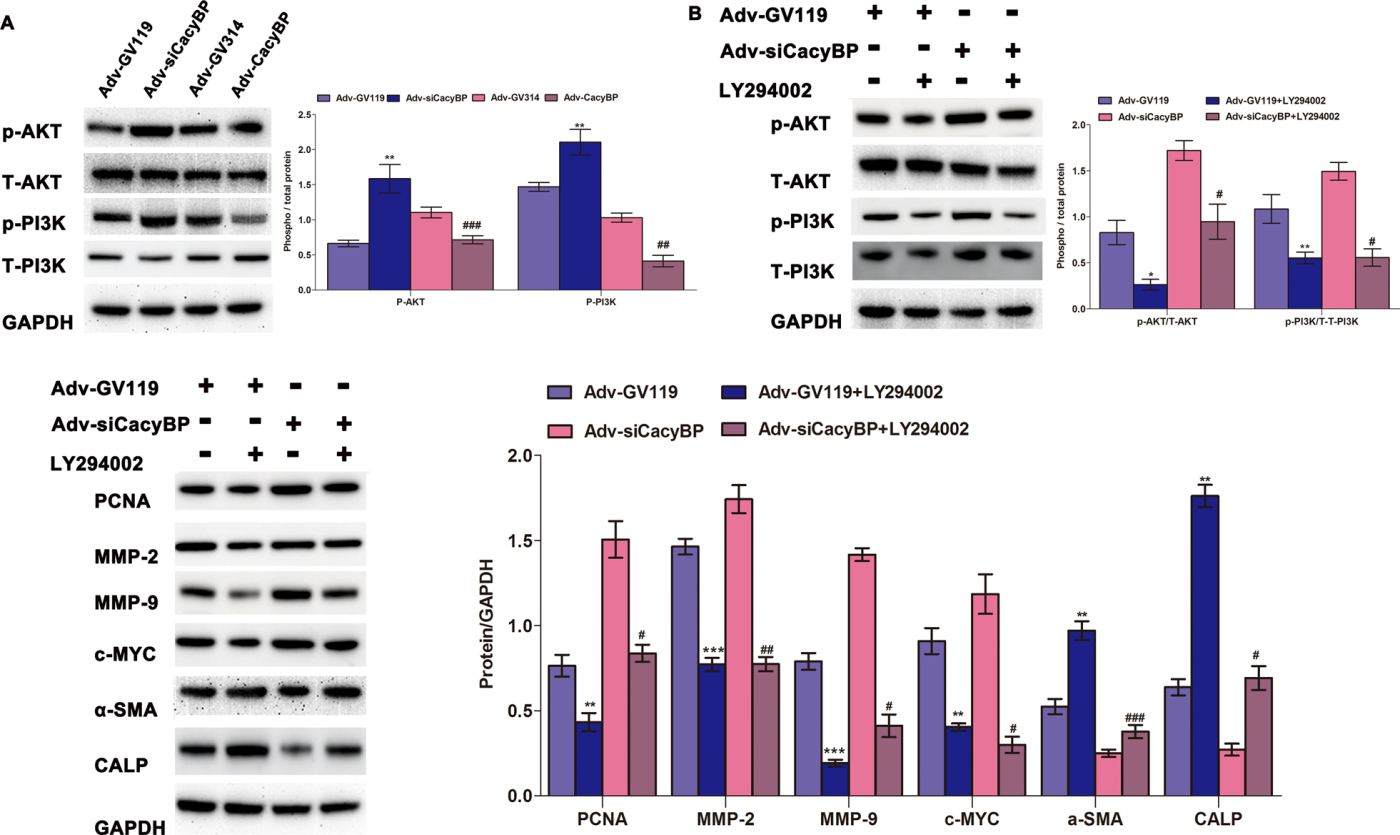
CacyBP/SIP regulated hPASMC phenotypic switch via the PI3K/AKT signaling pathway. **a** Phosphorylation level of PI3K/AKT in hPASMCs among groups. Western blots were quantified by calculating the band density normalized to GADPH. The difference in the phosphorylation of each protein was compared between Adv-siCacyBP and Adv-GV119 groups, and between Adv-CacyBP and Adv-GV314 groups. *: Adv-siCacyBP vs. Adv-GV119; #: Adv-CacyBP vs. Adv-GV314. **P < 0.01; ##*P* < 0.01, ###*P* < 0.001. **b** The phosphorylation level of PI3K/AKT and expression of phenotype markers in hPASMCs after using the PI3K/AKT inhibitor LY294002 (30 nM). Western blots were quantified by calculating the band density normalized to GADPH. The difference was compared between Adv-GV119 + LY294002 and Adv-GV119 groups, and between Adv-siCacyBP + LY294002 and Adv-siCacyBP groups. *: Adv-GV119 + LY294002 vs Adv-GV119; #: Adv-siCacyBP + LY294002 vs Adv-siCacyBP. *P < 0.05, **P < 0.01, ***P < 0.001; #*P* < 0.05, ##*P* < 0.01, ###*P* < 0.001. For these analyses, data are shown as mean ± SEM (n = 3 for each group). *hPASMC* human pulmonary artery smooth muscle cell, *CacyBP/SIP* calcyclin-binding protein, *PI3K* phosphoinositide 3-kinase, *AKT* protein kinase B

The PI3K/AKT signaling pathway may be involved in the process of cytoskeleton rearrangements and phenotypic switching of hPASMCs. We showed that the phosphorylation level of PI3K p85 (Tyr458)/p55 (Tyr199) and AKT (Ser473) increased significantly in hPASMCs upon lower CacyBP/SIP expression, whereas it was reduced upon CacyBP/SIP overexpression (Fig. 5A). The PI3K/AKT inhibitor LY294002 was used to block this signaling pathway to explore changes in hPASMC dysfunction induced by low CacyBP/SIP levels. As shown in Fig. 5B, inhibition of the PI3K/AKT pathway by LY294002 rescued the adverse phenotypic switch in hPASMCs induced by the decrease of CacyBP/SIP expression.

### Decrease of CacyBP/SIP expression promoted pulmonary vascular remodeling

**Fig. 6:**
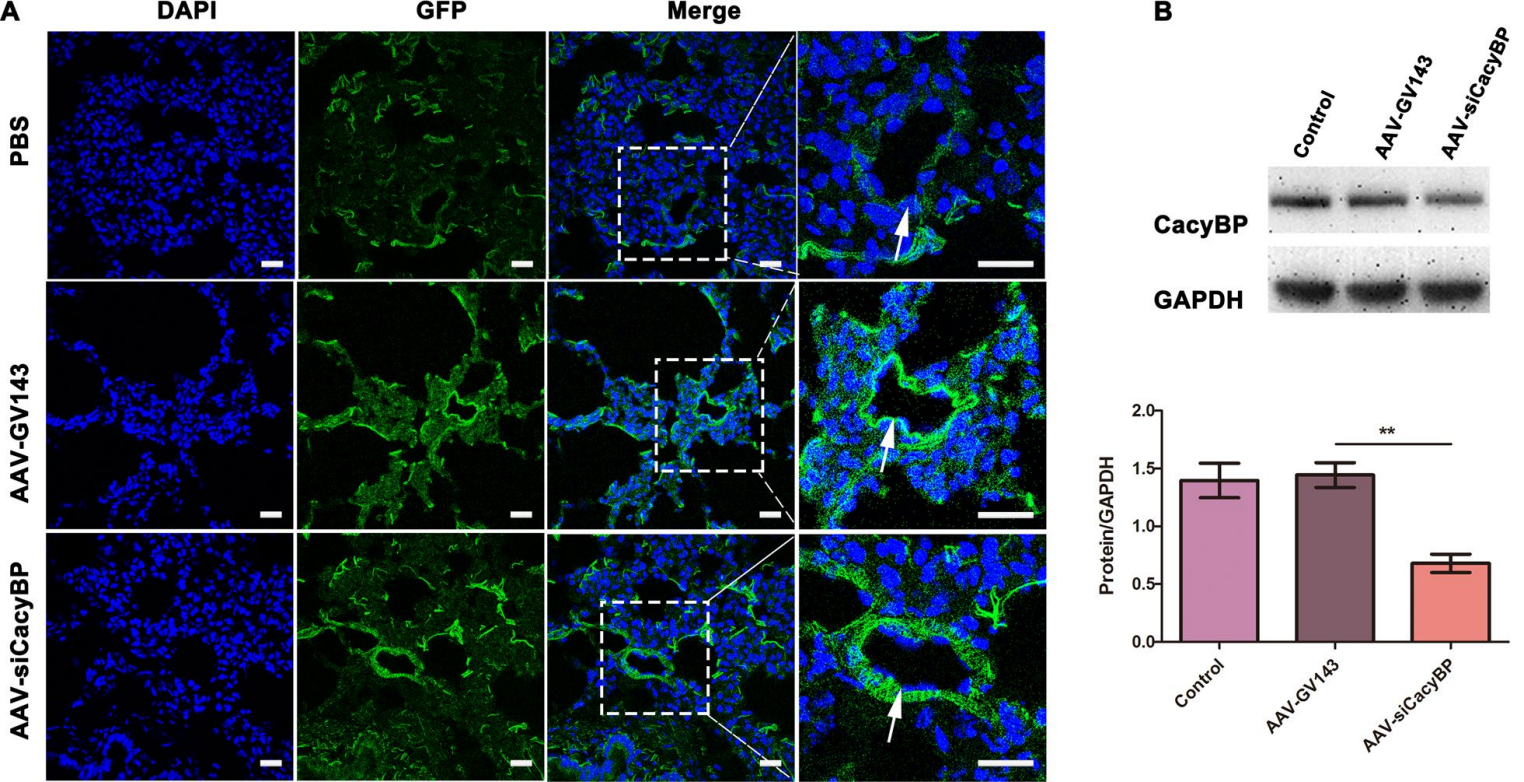
CacyBP/SIP expression was decreased in the pulmonary arterioles of rats after transduction with AAVs. **a** Representative images of the pulmonary arterioles after transduction with AAVs that contained the gene encoding for the green fluorescent protein. Scale bars, 25 µm. **b** Expression of CacyBP/SIP in the lung tissues of rats transduced with AAVs. Western blots were quantified by calculating the band density normalized to GADPH. Data are shown as mean ± SEM (n = 3 for each group). **P < 0.01. *AAVs* adeno-associated viruses, *CacyBP/SIP* calcyclin-binding protein

**Fig. 7:**
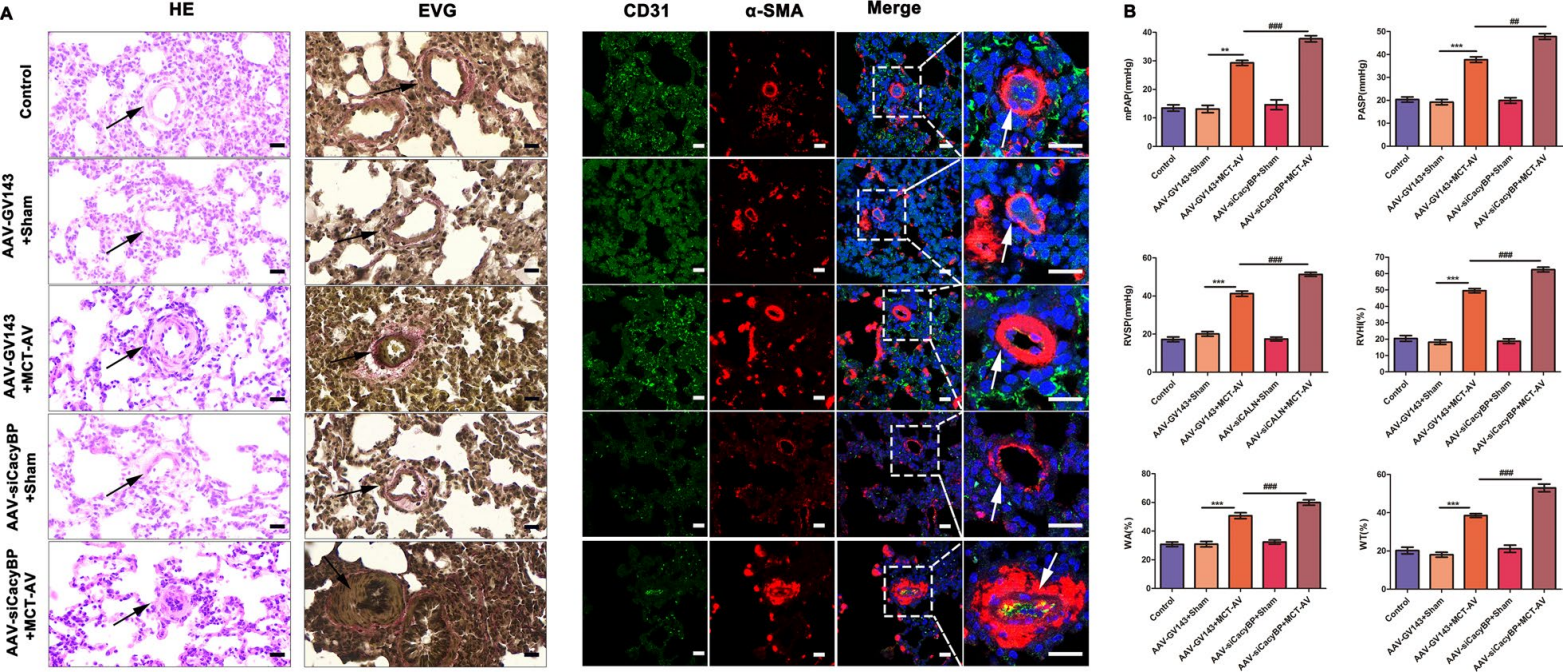
Decrease in CacyBP/SIP expression in rats aggravated flow-associated PAH and vascular remodeling. **a** H&E, EVG, and IF showing morphological changes of the pulmonary vascular remodeling in the indicated groups. Scale bars (black: 20 µm; white: 25 µm). **b** Quantification of hemodynamic and morphological parameters in the indicated groups. The hemodynamic data were recorded and analyzed using the PowerLab data acquisition system. mPAP, PASP, and RVSP of rats were calculated at least five respiratory cycles. Peripheral vessels 50–150 µm in diameter were randomly chosen and examined for morphological analysis. WT% and WA% of 10–15 arterioles were calculated per rat (WT% = [(external diameter–internal diameter)/external diameter × 100]; WA% = [(total area–luminal area)/total area × 100]). RVHI was calculated as the weight ratio of RV/(left ventricle plus atrial ventricular septum). Data are shown as the mean ± SEM (n = 6 for each group). The difference in variables was compared between AAV-GV143 + MCT-AV and AAV-GV143 + sham groups, and between AAV-siCacyBP + MCT-AV and AAV-GV143 + MCT-AV groups. *: AAV-GV143 + MCT-AV vs. AAV-GV143 + sham; #: AAV-siCacyBP + MCT-AV vs. AAV-GV143 + MCT-AV. **P < 0.01, ***P < 0.001; ##*P* < 0.01, ###*P* < 0.001. *H&E* hematoxylin and eosin, *EVG* Elastic Van-Gieson, *IF* immunofluorescent, *AAVs* adeno-associated viruses, *CacyBP/SIP* calcyclin-binding protein, *MCT* monocrotaline, *AV* aortocaval, *PAH* pulmonary arterial hypertension, *mPAP* mean pulmonary arterial pressure, *PASP* pulmonary arterial systolic pressure, *RVSP* right ventricular systolic pressures, *WT%* ratio of wall thickness, *WA%* ratio of wall area, *RVHI* right ventricular hypertrophy index

**Fig. 8:**
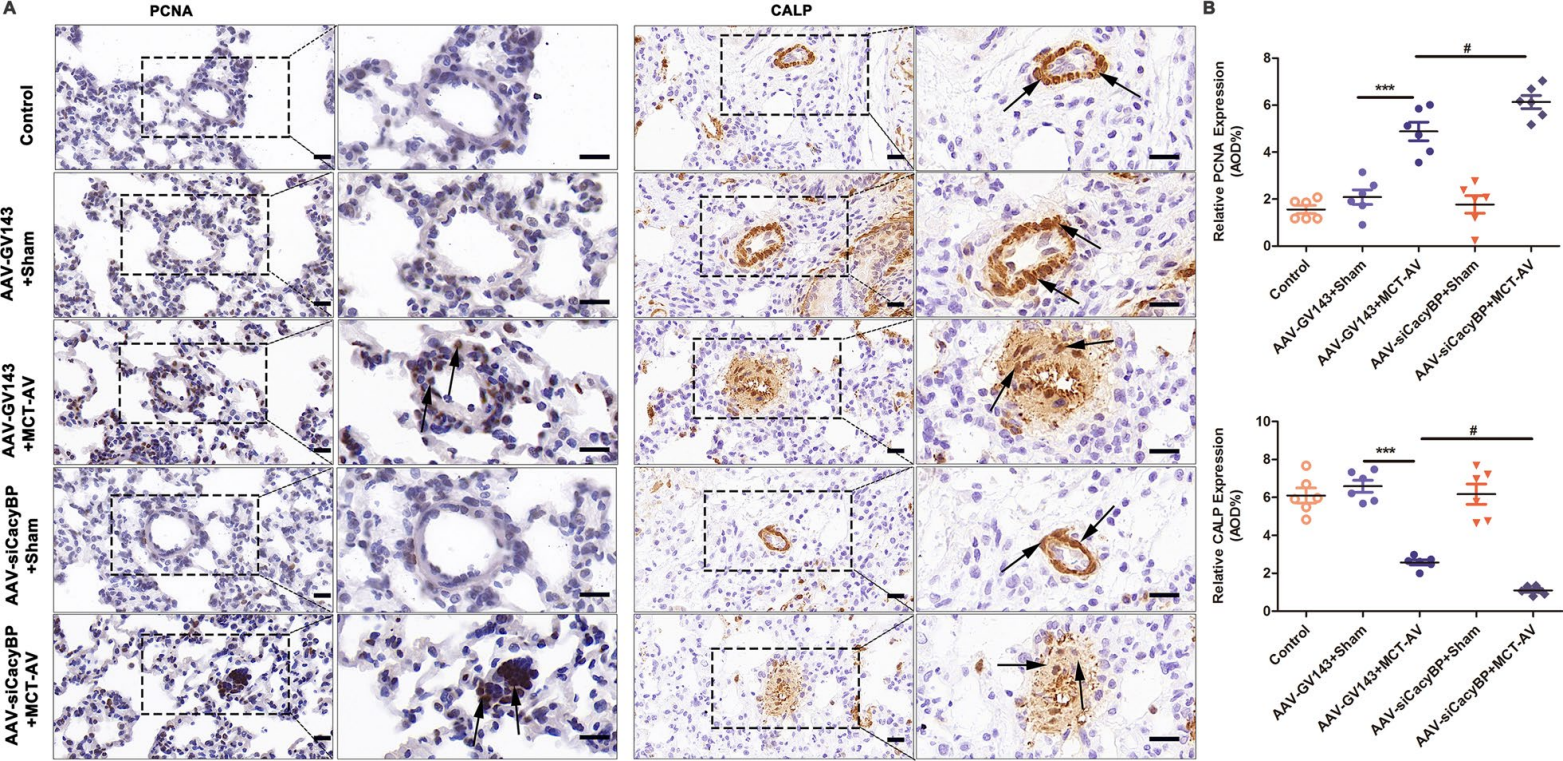
Decrease in CacyBP/SIP expression resulted in increased PCNA and reduced CALP expression. **a** Representative IHC images showing expression of PCNA and CALP in the pulmonary arterioles in the indicated groups. Scale bars, 20 µm. **b** Quantification of the expression of PCNA and CALP in lung tissues. AOD, calculated as the integrated option density/area, was used to quantify expression of PCNA and CALP. Data are shown as mean ± SEM (n = 6 for each group). The difference was compared between AAV-GV143 + MCT-AV and AAV-GV143 + sham groups, and between AAV-siCacyBP + MCT-AV and AAV-GV143 + MCT-AV groups. *: AAV-GV143 + MCT-AV vs. AAV-GV143 + sham; #: AAV-siCacyBP + MCT-AV vs. AAV-GV143 + MCT-AV. ***P < 0.001; #*P* < 0.05. *IHC* immunohistochemistry, *AAVs* adeno-associated viruses, *CacyBP/SIP* calcyclin-binding protein, *CALP* calponin, *MCT* monocrotaline, *AV* aortocaval, *PAH* pulmonary arterial hypertension, *AOD* average optical density

On the basis of our findings that (1) CacyBP/SIP levels decreased in the pulmonary arterioles of patients with CHD-PAH and in flow-associated PAH rats, and (2) low CacyBP/SIP expression induced the phenotypic switch and excessive proliferation and migration of hPASMCs, we hypothesized that decreased CacyBP/SIP expression may promote vascular remodeling in PAH. To address this hypothesis, AAV-siCacyBP was used to suppress expression of CacyBP/SIP in the pulmonary arterioles of flow-associated PAH rats. Strong green fluorescence was observed in the pulmonary arterioles of rats injected with AAVs (Fig. 6A), and protein semi-quantification confirmed that AAVs effectively reduced CacyBP/SIP expression in rat lung tissues (Fig. 6B). As shown in Fig. 7, after CacyBP/SIP inhibition, the degree of muscularization of small pulmonary arterioles in flow-associated PAH rats increased, and was accompanied by higher RVHI and worse hemodynamics including elevated RVSP, PASP, and mPAP compared to that in the MCT-AV group. Moreover, the expression of PCNA increased and that of CALP decreased in AAV-siCacyBP PAH rats, thus accounting for the severe pulmonary vascular remodeling (Fig. 8).

## Discussion

PASMCs in PAH display neoplastic-like characteristics, including proliferative and pro-migratory phenotypes, resulting in abnormal remodeling of the pulmonary arteries. In our study, we revealed the role of CacyBP/SIP in CHD-PAH. CacyBP/SIP expression in PASMCs was significantly decreased in remodeled pulmonary arterioles. Moreover, CacyBP/SIP levels were negatively correlated with pulmonary vascular pathology. We found that decreased CacyBP/SIP expression modulated the phenotypic switch of PASMCs via the PI3K/AKT signaling pathway to promote established PAH and related vascular remodeling. We highlight the significance of CacyBP/SIP in the pathogenesis of CHD-PAH, suggesting its potential role in disease amelioration.

Previous studies have shown that CacyBP/SIP was essential for the regulation of a broad spectrum of physiological and pathological processes. Specifically, CacyBP/SIP was mainly involved in cell proliferation [8], differentiation [9, 23], and ubiquitination [24], and its dysfunction was associated with development of various tumors [10, 25]. Several studies have shown that CacyBP/SIP was notably overexpressed in pancreatic, colorectal, and breast cancer [14–16], while it was strongly decreased in gastric and renal carcinoma, where it was considered as a tumor suppressor [12, 13]. Therefore, the function of CacyBP in the regulation of various diseases is controversial. PAH, also considered as “cardiovascular cancer,” shares similar underlying mechanisms with cancer, including uncontrolled cell proliferation, and is characterized by pulmonary vascular remodeling. Our results provided evidence that expression of CacyBP/SIP decreased in hPASMCs of CHD-PAH patients and in MCT-AV rats, suggesting that downregulation of CacyBP/SIP was involved in the dysfunction of PASMCs.

Similar to previous findings in renal, gastric, and glioma carcinoma cells [12, 13, 24], CacyBP/SIP inhibited the phenotypic switch in PASMCs, and prevented excessive proliferation and migration, while decreased CacyBP/SIP expression triggered the opposite effects. However, several studies have also elucidated the proliferative and pro-migratory properties of CacyBP/SIP in colon and pancreatic cancer cells [8, 16, 26, 27]. These conflicting results have raised confusion regarding the differential regulatory roles of CacyBP/SIP in diseases. We assumed that this may be due to cell type-specific functions of CacyBP/SIP, although the underlying mechanisms require further exploration.

PI3K/AKT plays a vital role in the regulation of cellular functions, including cell metabolism, growth, proliferation, survival, transcription, and protein synthesis [28]. Activation of the PI3K/AKT pathway promoted proliferation and migration of PASMCs, which is a well-recognized characteristic of PAH [29–32]. Decrease of CacyBP/SIP effectively increased the phosphorylation levels of PI3K and AKT, resulting in a change in the phenotypic switch of PASMCs. However, after inhibiting the PI3K/AKT pathway, the effects of CacyBP/SIP in hPASMCs were abrogated, suggesting that CacyBP/SIP was involved in the phenotypic switch, proliferation, and migration of hPASMC via this pathway. Nevertheless, the exact mechanism of PI3K/AKT activation in hPASMCs induced by CacyBP/SIP remains unknown. Notably, the phenotypic switch of PASMCs, characterized by changes in smooth muscle-specific gene expression, contributes to vascular remodeling in PAH. Previous studies have demonstrated that the transcription of smooth muscle-specific genes was regulated by cytoskeletal rearrangement [33]. Fan et al. [29] confirmed that inhibition of PI3K blocked AKT phosphorylation and attenuated the dysfunction of cytoskeletal rearrangements, suggesting a vital role for PI3K/AKT in the modulation of cytoskeleton rearrangements and phenotype switching, which are necessary for cell motility. Moreover, CacyBP/SIP was found to interact with tubulin, actin, and tropomyosin, thus regulating the organization and functional properties of microtubules, F-actin, and thin filaments [34–36]. Therefore, as PI3K/AKT and CacyBP/SIP both play a role in cytoskeletal rearrangement, we hypothesized that the level of CacyBP/SIP may correlate with, rather than cause, PI3K/AKT activation. Further studies are required to validate this.

In addition, some studies have reported that tumors can cause changes in healthy tissues adjacent to cancerous tissues. Studies have shown that cancer alters whole-body metabolism [37] and can induce complex DNA damage in distant tissues [38]. This indicates that the lung tissues used as control may have influenced the results of our study. However, the tissues obtained from cancer patients were merely used for preliminary exploration of CacyBP/SIP expression with IHC and IF. The differences in CacyBP expression between CHD-PAH patients and controls were obvious, and similar alterations were confirmed in subsequent cell culture and animal experiments, suggesting that our results are reliable. Our findings provide novel molecular insights into the PASMC phenotypic switch, excessive proliferation, and migration. We suggest that CacyBP/SIP may potentially reverse pulmonary vascular remodeling, and may serve as a novel target to ameliorate CHD-PAH.

## Conclusions

CacyBP/SIP expression was decreased in the remodeled pulmonary arteries, and correlated with the pathological grade of pulmonary arterioles in CHD-PAH patients. CacyBP/SIP protected against dysfunction of PASMCs via the PI3K/AKT signaling pathway, while restoration of CacyBP/SIP expression reversed pulmonary vascular remodeling in CHD-PAH. Our study revealed a novel role for CacyBP/SIP as a potential therapeutic target for CHD-PAH.

## Supplementary Information

**Additional file 1: Table S1.** Antibodies information for immunohistochemistry (IHC), immunofluorescent (IF) and western blot.

## Data Availability

The datasets used and/or analyzed during the current study are available from the corresponding author upon reasonable request.

## Abbreviations

PAH: Pulmonary arterial hypertension
CHD: Congenital heart disease
CHD-PAH: Pulmonary arterial hypertension associated with congenital heart disease
CacyBP: Calcyclin-binding protein
SIP: Siah-1 interacting protein
PASMCs: Pulmonary artery smooth muscle cells
hPASMCs: Human pulmonary artery smooth muscle cells
AAV: Adeno-associated viruses
IF: Immunofluorescent
IHC: Immunohistochemistry
α-SMA: Alpha smooth muscle actin
CALP: Calponin
MMP: Matrix metalloproteinase
RHC: Right heart catheterization
mPAP: Mean pulmonary artery pressure
RVSP: Right ventricular systolic pressure
PASP: Pulmonary arterial systolic pressure
MCT: Monocrotaline
AV: Aortocaval
RVHI: Right ventricular hypertrophy index
RV: Right ventricle
LV + S: Left ventricle plus atrial ventricular septum
HE: Hematoxylin and eosin
EVG: Elastic Van-Gieson
WT%: Wall thickness/vascular external diameter
WA%: Vascular area/total vascular area
PCNA: Proliferating cell nuclear antigen
NC: Negative control
PI3K: Phosphatidylinositol-3-kinases
AKT: Protein-serine-threonine kinase
