# Supplementary material for: Protective effects of calcyclin-binding protein against pulmonary vascular remodeling in flow-associated pulmonary arterial hypertension
Source: Respir Res. 2022 Aug 30;23:223. doi: 10.1186/s12931-022-02137-z (PMC9429705; doi:10.1186/s12931-022-02137-z)
Supplement: Supplementary file 1 — Additional file 1: Table S1. Antibodies information for immunohistochemistry (IHC), immunofluorescent (IF) and western blot. [file 12931_2022_2137_MOESM1_ESM.docx]

**Additional file 1. Antibodies information for immunohistochemistry (IHC), immunofluorescent (IF) and western blot.**

| Primary antibodies | Supplier | Catalog number | Species | Type | Dilution (IHC) | Dilution (IF) | Dilution (western blot) |
| --- | --- | --- | --- | --- | --- | --- | --- |
| CacyBP/SIP | Abcam | ab171972 | Rabbit | Monoclonal | 1:100­­­­ | 1/100 | 1:10000­­­­ |
| CD31 | Abcam | ab24590 | Mouse | Monoclonal |  | 1:100­­­­ |  |
| a-SMA | Abcam | ab7817 | Mouse | Monoclonal | | 1:400­­­­ | 1:800­­­­ |
| PCNA | Abcam | ab92552 | Rabbit | Monoclonal | 1:2000­­­ |  | 1:1000­­­­ |
| CALP | Abcam | ab46794 | Rabbit | Monoclonal | 1:1000­­­­ |  | 1:1000­­­­ |
| MMP-2 | Abcam | ab92536 | Rabbit | Monoclonal |  |  | 1:1000­­­­ |
| MMP-9 | Abcam | ab76003 | Rabbit | Monoclonal |  |  | 1:1000­­­­ |
| c-MYC | Abcam | ab32072 | Rabbit | Monoclonal |  |  | 1:1000­­­­ |
| p-AKT | CST | 9271 | Rabbit | Monoclonal |  |  | 1:1000­­­­ |
| AKT | CST | 9272 | Rabbit | Monoclonal |  |  | 1:1000­­­­ |
| p-PI3K | CST | 4228 | Rabbit | Monoclonal |  |  | 1:1000­­­­ |
| PI3K | CST | 4292 | Rabbit | Monoclonal |  |  | 1:1000­­­­ |
| GAPDH | CST | 5174 | Rabbit | Monoclonal |  |  | 1:1000­­­­ |
